# Evaluation of the Finis Swimsense^®^ and the Garmin Swim^™^ activity monitors for swimming performance and stroke kinematics analysis

**DOI:** 10.1371/journal.pone.0170902

**Published:** 2017-02-08

**Authors:** Robert Mooney, Leo R. Quinlan, Gavin Corley, Alan Godfrey, Conor Osborough, Gearóid ÓLaighin

**Affiliations:** 1 Electrical & Electronic Engineering, School of Engineering & Informatics, NUI Galway, Galway, Ireland; 2 Bioelectronics Research Cluster, National Centre for Biomedical Engineering Science, NUI Galway, Galway, Ireland; 3 Physiology, School of Medicine, NUI Galway, Galway, Ireland; 4 CÚRAM (SFI Centre for Research in Medical Devices), NUI Galway, Galway, Ireland; 5 Institute for Ageing and Health, Newcastle University, Newcastle upon Tyne, United Kingdom; 6 Department of Exercise & Sports Science, Manchester Metropolitan University. Crewe, United Kingdom; Nanyang Technological University, SINGAPORE

## Abstract

**Aims:**

The study aims were to evaluate the validity of two commercially available swimming activity monitors for quantifying temporal and kinematic swimming variables.

**Methods:**

Ten national level swimmers (5 male, 5 female; 15.3±1.3years; 164.8±12.9cm; 62.4±11.1kg; 425±66 FINA points) completed a set protocol comprising 1,500m of swimming involving all four competitive swimming strokes. Swimmers wore the Finis *Swimsense* and the Garmin *Swim* activity monitors throughout. The devices automatically identified stroke type, swim distance, lap time, stroke count, stroke rate, stroke length and average speed. Video recordings were also obtained and used as a criterion measure to evaluate performance.

**Results:**

A significant positive correlation was found between the monitors and video for the identification of each of the four swim strokes (Garmin: *X*^2^ (3) = 31.292, p<0.05; Finis:*X*^2^ (3) = 33.004, p<0.05). No significant differences were found for swim distance measurements. Swimming laps performed in the middle of a swimming interval showed no significant difference from the criterion (Garmin: bias -0.065, 95% confidence intervals -3.828–6.920; Finis bias -0.02, 95% confidence intervals -3.095–3.142). However laps performed at the beginning and end of an interval were not as accurately timed. Additionally, a statistical difference was found for stroke count measurements in all but two occasions (p<0.05). These differences affect the accuracy of stroke rate, stroke length and average speed scores reported by the monitors, as all of these are derived from lap times and stroke counts.

**Conclusions:**

Both monitors were found to operate with a relatively similar performance level and appear suited for recreational use. However, issues with feature detection accuracy may be related to individual variances in stroke technique. It is reasonable to expect that this level of error would increase when the devices are used by recreational swimmers rather than elite swimmers. Further development to improve accuracy of feature detection algorithms, specifically for lap time and stroke count, would also increase their suitability within competitive settings.

## Introduction

Swimming ranks amongst the most popular leisure activities worldwide [1,2]. The general health benefits of regular swimming are well established and swimming is one of the few sports that can be enjoyed during all stages of life [3]. Individuals who swim as a recreational activity for health and fitness can benefit from monitoring some basic indices of their performance. Parameters may include the time or distance completed; in much the same fashion as a recreational runner will use a stopwatch or GPS device. Indeed, research evidence suggests that better health outcomes can arise when levels of physical activity are quantified [4].

Additional benefits of quantifying swimming performance for health may include assisting with goal-setting, as an activity diary, as a means of monitoring trends in performance over time or as a motivational tool. In aquatic settings such variables would typically be measured using manual methods such as a stopwatch. However, manual methods are prone to inconsistencies and inaccuracies. Furthermore, recreational swimmers do not typically have the availability of a coach or other observer who can record this information for them, using video for example [5].

Wearable sensor technologies have gained popularity in many sporting settings and commercially available products have been validated for use across a range of physical activities [6–8]. With advances in MEMS-based kinematic sensing, swimmers can also now monitor their own activity in their normal training environment using wearable technologies [9]. Several prototype designs have been described and validated in the swimming literature [10–13]. Additionally, commercially available swimming activity monitors have gained prominence, including the Finis *Swimsense*^®^ (FINIS USA, Livermore, CA, USA.) and Garmin *Swim*^*TM*^ (Garmin International Inc, Olathe, KS, USA.).

These commercial activity monitors include features such as stroke counting and swim speed measurements and can identify the different strokes performed automatically. Feedback is provided either instantly on the wrist worn interface or by downloading the data to custom designed websites for a more detailed analysis once the swimming session has been completed. These devices are marketed directly at the swimmer and are primarily aimed for recreational, self-coached and amateur swimmers or triathletes as opposed to elite swimmers. These systems are seldom used by swim coaches for competitive swimming training and performance analysis [14]. These activity monitors offer significant potential in recreational swimming settings by providing swimmers with a method of quantifying and analysing their own training in the pool. However, to the authors’ knowledge, these devices have not yet received objective scrutiny to validate their performance. The activity monitors are designed for people who train in order to achieve personal swim training goals.

The aim of this paper is to assess the accuracy of the Finis *Swimsense* and the Garmin *Swim* activity monitors in providing accurate feedback on a range of swimming performance parameters for each of the four competitive swimming strokes.

## Methods

### Participants

Ten national level competitive swimmers were recruited to take part in the study (5 male, 5 female; 15.3 ±1.3 years; 164.8 ±12.9 cm; 62.4 ±11.1 kg; 425 ±66 FINA points (Fédération internationale de natation)). Competitive athletes were chosen over recreational swimmers in order to ensure that the participants would be fully competent in performing all four competitive swimming strokes in a highly consistent manner over the protocol distance. In doing so, it was expected to achieve the absolute best estimate of accuracy that could be attained from the activity monitors in a recreational setting. The study received approval (reference number 13/NOV/08) from the institutional ethics committee, NUI Galway Research Ethics Committee (REC), and followed the terms of the Declaration of Helsinki. The protocol was explained to the swimmers and their parents. Parental written consent was obtained and the participants provided written informed assent.

### Procedures

Data collection took place in a temperature controlled 25 m indoor swimming pool (water temperature 29°C), which was within the normal operating temperature for both swim monitors. Participants were fitted with a monitor on each wrist, which was allocated at random. Both devices feature tri-axial accelerometers to automatically track the acceleration of the wrist as the swimmer moves through the water. Pool length can be readily adjusted on both devices and was programmed to suit the 25 m environment. Settings were configured for each individual user (height, mass, age, wrist used) and the participants completed a self-directed warm up of 15 minutes duration to prepare physically and to habituate to wearing the devices whilst swimming.

Participants were instructed to complete a swimming session totalling 1,500 m (60 laps) comprising each of the four competitive swimming strokes, completed in individual medley order (i.e. butterfly, backstroke, breaststroke, frontcrawl). Butterfly was swum in 50 m intervals followed by 45 s rest, repeated six times. The other strokes were swum in 100 m intervals, again followed by 45 s rest, repeated four times. Two minutes of rest was included when transitioning between strokes, during which swimmers were instructed to remain still with their forearms resting on the pool deck. In total, 15,000 m of swimming were completed, generating 600 laps, or data sets, for statistical analysis. Swimming speed was self-selected during all trials.

Trials were simultaneously captured at 50 Hz using two fixed underwater cameras (GoPro Hero3+) positioned to record all events occurring at the pool walls in order to identify wall contact events and one panning video camera on the pool deck to record the participants throughout each lap (Sony Handycam HDR-XR550). Images from the three cameras were synchronised by interpolating the data according to the time lag between cameras using a blinking light source [15]. Video footage was subsequently used as the criterion measure to assess the performance of the swim activity monitors.

### Data processing & analysis

Video files were stored on a portable hard drive and analysed with the use of Dartfish Video Software (ProSuite version 5.5; Dartfish, Fribourg, Switzerland) to allow for criterion measures of all variables to be determined through manual observation of the video footage.

Inter-operator and intra-operator reliability testing was carried out by calculating the intra-class correlation coefficient (ICC) on a segment of the video data for lap time and stroke count. ICC is used to interpret the relationship between two variables that record the same measurement [16]. This was a necessary step in order to ensure the accuracy of the criterion measure. The other variables measured in the study can be derived from these variables so this was deemed sufficient for reliability assessment of the criterion measure. Intra-operator reliability for lap time (ICC = 0.999) and stroke count (ICC = 0.972) were found to be excellent. Inter-operator reliability for lap time (ICC = 0.993) and stroke count (ICC = 1.000) were also found to be excellent [17]. These results indicate that the video footage is a valid criterion measure from which to compare the performance of the activity monitors.

Data from each activity monitor were downloaded and exported to Microsoft Excel (2010 version; Microsoft, USA) for collation and processing. Stroke type, swim distance, lap time, stroke count, and average speed were measured on both activity monitors. Additionally, stroke rate and stroke length were also recorded for the Finis *Swimsense*. These were not available features on the Garmin *Swim*.

Descriptive statistics (mean, standard deviation) were determined for all variables. The Kolmogorov-Smirnov test was used to assess if the data were parametric or non-parametric. Stroke identification data were categorical in nature and a Pearson’s chi-square test was used to assess for agreement between values [16].

Wilcoxon signed-rank tests were conducted to compare the relationship between non-parametric data.. The standard error of the mean was calculated to determine the standard deviation of the sample means. 95% limits of agreement were determined as the mean difference ±1.96 times the standard deviation of the difference. ICC were determined as a measure of the reliability of the devices. A linear mixed model was used to generate limits of agreement for each set of comparisons for the lap time and stroke count data which account for the (linked) replicates within individuals across devices [18,19]. These data (lap times and stroke counts) are the most critical and fundamental parameters measured here as these values are used in the determination of many of the other reported parameters. Data analyses were performed using Statistical Package for the Social Sciences for Windows (Version 21, SPSS Inc., Chicago, IL). A p-value of 0.05 was set for all statistical analyses.

## Results

Table 1 compares the sensitivity and specificity of the stroke type identification function for both activity monitors. The Garmin *Swim* correctly identified which of the four competitive swimming strokes was performed for a given lap with 95.4% overall sensitivity rate whilst the Finis *Swimsense* was slightly more sensitive at 96.4% overall. It was also found that there was a significant correlation in stroke type identification between the activity monitors and video for each of the four strokes (Garmin: *X*^2^ (3) = 31.292, p<0.05; Finis: *X*^2^ (3) = 33.004, p<0.05). Taking each stroke in isolation, a sensitivity of 94% or greater was achieved in all but two cases; namely breaststroke when recorded with the Garmin (86.0%) and backstroke when recorded by the Finis monitor (88.9%). This is also reflected in the slightly lower specificity values for these two strokes.

**Table 1:** Sensitivity and specificity of stroke identification for Finis *Swimsense* and Garmin *Swim*. The actual stroke completed for each lap was compared against the success of the sensors to correctly identify each lap. For both devices, a significant association was found with the actual stroke completed. Sensitivity is a measure of the proportion of positives that are correctly identified, whilst specificity measures the proportion of negatives that are correctly identified. (Fly = Butterfly; Bk = Backstroke; Brs = Breaststroke; Fc = Frontcrawl; Miss = no lap registered).

	Sensitivity					Specificity
**Garmin**	Fly	Bk	Brs	Fc	Miss	
Fly	**94.9%**	0%	0.8%	4.2%	0%	100.0%
Bk	0%	**98.8%**	0%	1.3%	0%	95.8%
Brs	0%	13.2%	**86.0%**	0.7%	0%	99.8%
Fc	0%	0%	0%	**98.3%**	1.7%	98.1%
**Finis**	Fly	Bk	Brs	Fc	Miss	
Fly	**97.2%**	0%	0%	0.9%	1.9%	100.0%
Bk	0%	**88.9%**	10.4%	0%	0.7%	99.8%
Brs	0%	0.8%	**99.2%**	0%	0%	96.5%
Fc	0%	0%	0%	**100.0%**	0%	99.7%

The total distance recorded by each sensor was compared to the actual total distance completed. Both activity monitors performed with very high accuracy when measuring the total distance completed for all four swimming strokes. A cumulative total of 15,000 m was completed by the participants. The Garmin monitor registered a total of 14,925 m (99.5% detection accuracy), which was 75 m, or three laps, short. These missed laps were all for the frontcrawl stroke. The Finis registered exactly 15,000 m correctly, however inspection of the results showed small variations within strokes (-1 lap butterfly; -3 laps backstroke +1 lap breaststroke; +3 laps frontcrawl, giving an adjusted detection accuracy of 98.7%).

Table 2 and Table 3 provide a comparison of performance of the activity monitors for other variables in the study. Lap times; stroke count; average speed, stroke rate and stroke length were statistically significantly different from the criterion measure in the majority of cases for both activity monitors and for all four strokes.

**Table 2:** Comparison of results for lap time and stroke count. Mean score, standard deviation (SD), standard error of the mean (SE), 95% confidence intervals, interclass correlation coefficient (ICC), limits of agreement (LOA), mean absolute percentage error (MAPE) and the error range are presented for both Finis *Swimsense* and Garmin *Swim* monitors and compared with the criterion measures extracted from video footage.

	Lap time (s)							Stroke count						
	Mean ±SD	SE	95% CI	ICC	LOA	MAPE (%)	Error Range (%)	Mean ±SD	SE	95% CI	ICC	LOA	MAPE (%)	Error Range (%)
**Butterfly**														
Video	20.31±2.46		(19.87–20.75)					12.2±1.8		(11.9–12.5)				
Garmin	23.33±3.49*	0.321	(22.70–23.96)	0.357	-8.293–5.199	17.2	(-13.6–114.5)	10.9±2.0*	0.183	(10.5–11.3)	0.295	-3.537–2.262	15.6	(-46.2–27.3)
Finis	23.30±5.28*	0.518	(22.29–24.31)	0.131	-7.046–4.521	20.6	(-20.7–117.7)	11.3±1.9*	0.186	(10.9–11.7)	0.758	-2.773–3.254	9.3	(-46.2–20.0)
**Backstroke**														
Video	22.38±1.38		(22.17–22.59)					8.9±1.1		(8.7–9.1)				
Garmin	23.93±3.24*	0.256	(23.43–24.43)	0.153	-8.293–5.199	11.6	(-22.4–74.1)	9.5±1.4*	0.110	(9.3–9.7)	0.453	-3.537–2.262	14.1	(-33.3–57.1)
Finis	23.64±2.91*	0.244	(23.16–24.12)	0.225	-7.046–4.521	9.7	(-18.6–69.4)	8.6±1.3	0.106	(8.4–8.8)	0.361	-2.773–3.254	12.0	(-40.0–62.5)
**Breaststroke**														
Video	25.23±2.05		(24.89–25.57)					9.9±1.6		(9.6–10.2)				
Garmin	26.59±3.10*	0.266	(26.07–27.11)	0.044	-8.293–5.199	11.2	(-28.1–48.9)	11.3±1.8*	0.152	(11.0–11.6)	0.542	-3.537–2.262	18.9	(-20.0–71.4)
Finis	26.48±3.07*	0.271	(25.95–27.01)	0.317	-7.046–4.521	9.7	(-20.7–47.3)	11.3±1.6*	0.145	(11.0–11.6)	0.650	-2.773–3.254	18.6	(-16.7–50)
**Frontcrawl**														
Video	21.24±1.76		(20.98–21.50)					9.3±1.2		(9.1–9.5)				
Garmin	22.33±5.18*	0.390	(21.56–23.10)	0.117	-8.293–5.199	8.4	(-24.2–50.6)	9.4±2.5	0.192	(9.0–9.8)	0.169	-3.537–2.262	11.4	(-50.0–58.3)
Finis	21.82±2.68*	0.202	(21.42–22.22)	0.635	-7.046–4.521	7.7	(-40.3–57.3)	9.8±1.3*	0.096	(9.6–10.0)	0.062	-2.773–2.262	14.4	(-44.4–62.5)

Values denoted with an asterisk (*) indicate that a significant difference exists between the sensor device and the criterion (p<0.05).

**Table 3:** Comparison of results for stroke rate, stroke length and average speed. Mean score, standard deviation (SD), standard error of the mean (SE), 95% confidence intervals, interclass correlation coefficient (ICC), mean absolute percentage error (MAPE) and the error range are presented for both Finis *Swimsense* and Garmin *Swim* monitors, where applicable and compared with the criterion measures extracted from video footage.

	Stroke rate (str/min)						Stroke length (m)						Average speed (m/s)					
	Mean ±SD	SE	95% CI	ICC	MAPE (%)	Error Range (%)	Mean ±SD	SE	95% CI	ICC	MAPE (%)	Error Range (%)	Mean ±SD	SE	95% CI	ICC	MAPE (%)	Error Range (%)
**Butterfly**																		
Video	46.1±5.4		(45.1–47.1)				1.64±0.18		(1.61–1.67)				1.24±0.14		(1.21–1.27)			
Garmin	N/A	N/A	N/A	N/A	N/A	N/A	N/A	N/A	N/A	N/A	N/A	N/A	1.09±0.15*	0.014	(1.06–1.12)	0.327	13.2	(-53.5–16.5)
Finis	36.0±9.2*	0.903	(34.2–37.8)	0.135	20.8	(-73.0–6.2)	2.29±0.47*	0.046	(2.20–2.38)	0.127	40.9	(0.9–138.9)	1.12±0.20*	0.020	(1.08–1.16)	0.068	16.4	(-53.8–31.5)
**Backstroke**																		
Video	29.5±3.7		(28.9–30.1)				2.31±0.22		(2.27–2.35)				1.12±0.07		(1.11–1.13)			
Garmin	N/A	N/A	N/A	N/A	N/A	N/A	N/A	N/A	N/A	N/A	N/A	N/A	1.06±0.13*	0.011	(1.04–1.08)	0.198	10.0	(-42.5–29.2)
Finis	24.0±4.1*	0.346	(23.3–24.7)	0.168	18.0	(-60.8–4.5)	2.95±0.46*	0.038	(2.87–3.03)	0.093	30.4	(-51.9–119.8)	1.08±0.12*	0.010	(1.06–1.10)	0.286	8.2	(-40.9–22.9)
**Breaststroke**																		
Video	29.6±3.2		(29.0–30.2)				2.04±0.28		(1.99–2.09)				1.00±0.08		(0.99–1.01)			
Garmin	N/A	N/A	N/A	N/A	N/A	N/A	N/A	N/A	N/A	N/A	N/A	N/A	0.95±0.12*	0.011	(0.93–0.97)	0.076	10.4	(-73.0–39.1)
Finis	27.1±3.3*	0.288	(26.5–27.7)	0.608	9.0	(-45.8–9.3)	2.26±0.33*	0.029	(2.20–2.32)	0.702	13.1	(-13.5–42.9)	0.97±.011*	0.010	(0.95–0.99)	0.286	8.6	(-31.1–36.2)
**Frontcrawl**																		
Video	33.4±4.4		(32.7–34.1)				2.15±0.21		(2.12–2.18)				1.19±0.10		(1.17–1.21)			
Garmin	N/A	N/A	N/A	N/A	N/A	N/A	N/A	N/A	N/A	N/A	N/A	N/A	1.10±0.20*	0.015	(1.07–1.13)	0.258	7.3	(-33.7–30.7)
Finis	28.9±3.9*	0.296	(28.3–29.5)	0.455	12.9	(-50.4–10.3)	2.59±0.39*	0.029	(2.53–2.65)	0.292	21.2	(-11.2–117.1)	1.17±0.17*	0.013	(1.14–1.20)	0.613	7.4	(-36.2–68.2)

Values denoted with an asterisk (*) indicate that a significant difference exists between the sensor device and the criterion (p<0.05).

**Fig 1:**
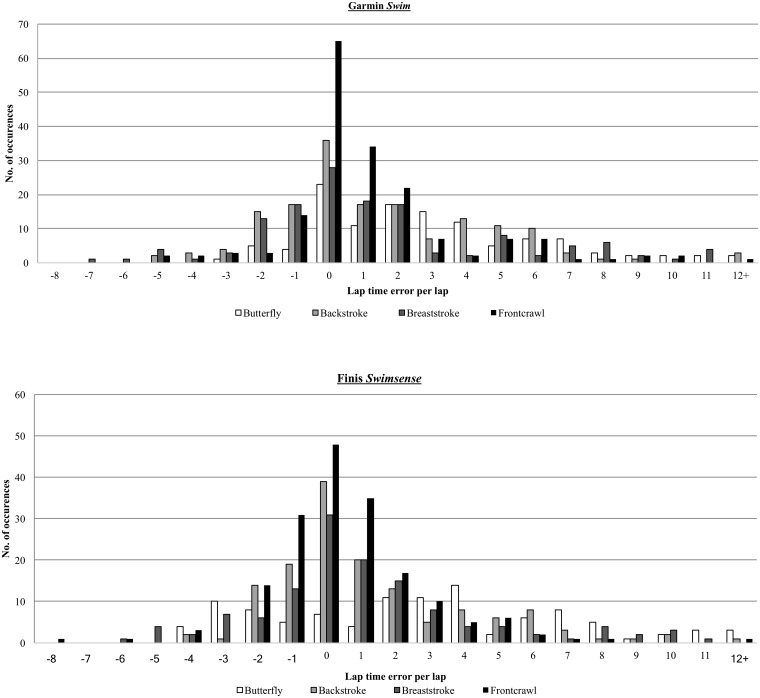
Comparison of overall frequency of error in the measurement of lap times for both Finis Swimsense and Garmin Swim.

A comparison of laps performed at the beginning of an interval (i.e. the first lap of four in a 100 m swim interval) were compared to those performed during the middle of an interval and those performed at the end of an interval. The butterfly trials were omitted from this analysis as butterfly was completed in 50 m intervals and thus did not include a middle lap for comparison. Both activity monitors demonstrated a similar pattern of error in lap times, with a statistically significance difference found for laps performed at the beginning and end of an interval but no statistical difference found for those performed in the middle of an interval (Fig 1). For example, the average front crawl mid interval lap time was 21.03 ±4.27 s. The Garmin *Swim* averaged 21.53 ±2.17 s (+2.4%) and the Finis *Swimsense* averaged 20.91 ±4.48 s (-0.6%). However for laps performed at the start and end of an interval, the reported error was much larger, ranging from -13.4% to +33.5%.

The results showed that mid interval lap times were accurately recorded, the Garmin *Swim* showed a bias of -0.065s, a lower limit of agreement of -3.828s and an upper limit of agreement of 6.920s. For the same laps, the Finis *Swimsense* demonstrated a bias of -0.02s, a lower limit of agreement of -3.095s and an upper limit of agreement of 3.142s. For starting laps, the results for Garmin showed a bias of 4.608s (-4.855s – 14.070s limits of agreement) and for Finis showed a bias of 3.84s (-5.199s – 12.871s). Finally, for end laps, the results for Garmin showed a bias of 1.382s (-4.157s – 6.920s) and for Finis showed a bias of 0.77s (-5.679–7.217s).

**Fig 2:**
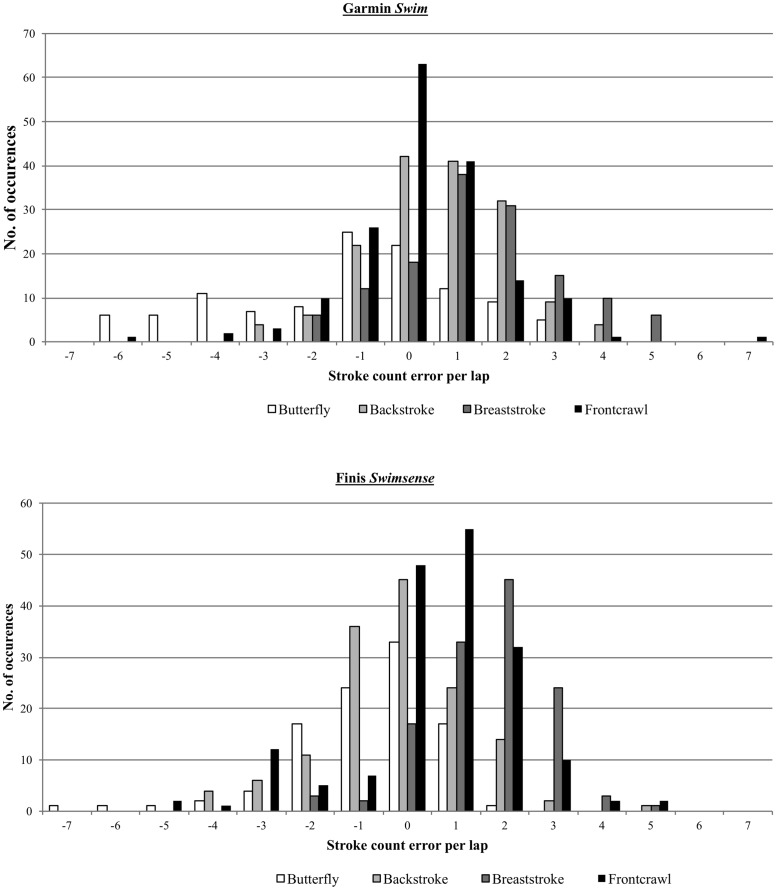
Comparison of overall frequency of error in the measurement of stroke count for both Finis Swimsense and Garmin Swim. The results indicate a significant overestimation of stroke count for both devices for all strokes except butterfly.

Fig 2 highlights the results of the stroke count measurements, demonstrating an overall overestimation of stroke count for both activity monitors. Taking all four strokes combined, the Finis monitor correctly registered the stroke count to within one stroke of the actual stroke count in 62.2% of laps. Similarly, the Garmin monitor was within one stroke of the actual stroke count in 62.5% of laps. Looking at each stroke in isolation, the trend towards overestimation of stroke count was observed in all strokes except butterfly, which showed a tendency towards underestimation for both activity monitors. The results for stroke count were statistically significantly different from the criterion measure in all but two instances; in backstroke for the Finis monitor and in frontcrawl for the Garmin monitor.

The highest level of accuracy for the Garmin monitor was found for frontcrawl, with the stroke count within one stroke of the actual stroke count in 75.6% of laps. With the Finis monitor, the highest level of accuracy was found in the backstroke (73.4% ±1 of actual). Breaststroke demonstrated the lowest stroke count accuracy for both devices (Finis 40.6% ±1 of actual; Garmin 50.0% ±1 of actual). For the Garmin monitor, the long axis strokes performed better than the short axis strokes, but this was not observed in the Finis monitor.

## Discussion

The aim of this study was to assess the accuracy of the Finis *Swimsense* and the Garmin *Swim* activity monitors and to assess the validity of using these devices in recreational settings. It is well established that the pattern of hand movement during swimming shows considerable variances owing to various factors including anthropometrics, skill level and fatigue [20–22]. With recreational swimmers, there can be a very wide variation in skill level and fatigue, with consequent high levels of variation in swim performance in this group of swimmers. Conversely, competitive athletes display more consistent patterns of movement [23] and thus these athletes were used for our testing in order to minimize variation in swimming performance. Thus the results obtained in this study would represent expected best case findings for these devices and it would be reasonable to expect that there would be a significant deterioration in the activity monitors’ performance when used by recreational swimmers.

When assessing the performance of these activity monitors it is important to consider carefully what can be regarded as an acceptable performance level for different categories of users. Whilst some findings in the present study suggest that some parameters were statistically significantly different from the criterion measures, these differences, in a sporting context, may or may not be at a scale to be of concern to the intended users of these activity monitors [24]. A table of proposed system requirements for swimming activity monitors when used by either recreational or competitive swimmers is presented in Table 4, showing that these two groups will have very different requirements for the accuracy of feedback information provided to them on their swimming performance.

**Table 4:** The system requirements of recreational and competitive swimmers will differ and have an impact on the level of accuracy required of the swimming monitors.

System Parameter	Recreational Swimmer	Competitive Swimmer
Lap time	Accuracy required to within ±2 seconds to monitor trends over time. A variance of 2 seconds over a lap time of 30 seconds equates to a 6.7% error.	Accuracy required to within ±0.3 seconds in order to be comparable with a stopwatch (current standard)
Stroke count	Accuracy within ±2 strokes sufficient to monitor trends over time	Accuracy required to lesson more than ±1 stroke per lap
Swim distance	Key determinant of training progression, accuracy required to within ±5% of actual (i.e. no more than 2 missed/additional laps included per 1,000 m completed in a 25 m pool)	Not applicable to user, training distances pre-prescribed and monitored by coach
Swim speed	Not applicable to user, lap times provide a sufficient metric	Accuracy within ±0.01 m/s required to relate to required lap time accuracy and also to compare with other reported methods. More concerned with instantaneous speed or speed during different race segments
Stroke rate	Not applicable to user, stroke counts provide a sufficient metric	Accuracy within ±5% adequate (i.e. ±2 str/min).More concerned with instantaneous stroke rate or stroke rate during different race segments
Stroke length	Accuracy within ±0.2 m sufficient and related to accuracy of stroke count measure	Accuracy close to 100% required (i.e. errors of no more than 0.1 m) and related to stroke rate measure. More concerned with stroke length during different race segments
Stroke identification	100% accuracy required as errors will be very apparent	100% accuracy required as errors will be very apparent

For example, a competitive swimmer or coach may require a lap time measure to be precise to within three tenths of a second in a training environment. This level of performance would effectively bridge the gap between the performance capabilities of a stopwatch and those of a video-based analysis system. The Garmin device registered the lap time to within 0.3 seconds on 18% of laps recorded. The Finis also showed a similar performance level (15%). The same could not be said for a recreational athlete, who would require a much less stringent level of lap time accuracy. Based on our experience working with both elite and recreational swimmers, lap time values of within one to two seconds of the actual time for a given lap would be appropriate for a recreational swimmer in order for them to gauge their performance level and to monitor gross improvements in performance over an extended period of time. In the present study, both devices registered the lap time within two seconds of the actual lap time on 67% of occasions.

Moreover, when measuring stroke count, recreational swimmers are likely to be more interested in monitoring the trends over a period of time, as opposed to monitoring the exact stroke count for each lap, in order to assess if training goals are being achieved and if swimming efficiency has improved. It was found that both devices registered the stroke count to within one of the actual stroke count on over 62% of laps recorded. In this context, both the Finis *Swimsense* and the Garmin *Swim* activity monitors would appear to provide recreational swimmers and triathletes, who without these types of devices, would not have a way of keeping a record of their training and progression. Conversely, a competitive swimmer will already have developed a consistent stroke count pattern through extensive training. These swimmers will have greater awareness of their stroke count for given laps and may deliberately make minor adjustments to their stroke count during training sets, in order to practice specific racing strategies for their different events, for example. As such, the stroke count accuracy would need to be very high for competitive swimmers.

The ability of such activity monitors to correctly identify the swimming stroke used in a given lap is a fundamental performance characteristic for monitoring both recreational and competitive activities. Notwithstanding the fact that the frontcrawl stroke may reasonably be assumed to be the most prevalent stroke in the majority of training settings, all four strokes may be used interchangeably during training, even for elite swimmers with specific stroke specializations. The results of the present study demonstrate that the Finis *Swimsense* performed slightly better than the Garmin *Swim*, but both sensors reported very high overall sensitivity and specificity for stroke identification (Table 1), which is comparable with previous research [10,25,26].

Closer inspection of the results in the present study suggests that where errors did occur these errors appear to be attributable to individual swimmers. For example, the Finis monitor registered an entire backstroke set for one swimmer as breaststroke, whilst the Garmin monitor incorrectly recorded breaststroke as backstroke on 14 of the 16 laps for another swimmer. However, as backstroke is performed in a supine position, in contrast to other strokes, it should be possible to correctly identify when this is being performed. It is conceivable that the misidentification issue could be linked to clockwise and counter-clockwise movements about the shoulder joint. Backstroke arm pull is opposite in direction to frontcrawl and butterfly, whilst breaststroke swimming has a more backward and forward movement of the wrist. Another possible explanation is that activity during rest periods, such as slight arm movements when standing at the pool wall, may lead to errors in the algorithm for stroke type identification.

This large level of misidentification could be due to individual variances in stroke technique. It is reasonable to expect that this level of misidentification would increase when the devices are used by recreational swimmers rather than elite swimmers.

Both activity monitors measured swim distance with excellent accuracy across all strokes. The swim distance is derived in both devices by multiplying the number of laps completed by the length of the pool. Therefore, swim distance is a function of the accuracy of the lap counter algorithm, which relies on accurate detection of wall contact events. Three types of wall contact events can be detected; those at the start and end of a swimming interval and those after turns. Data from a wrist worn accelerometer can be used to determine these events as a large impact acceleration peak will signify that a wall strike has occurred [27]. From a practical point of view, accurately recording the distance completed during a training activity is a fundamental function for recreational swimmers. In fact, this function may be used along with the total time spent swimming by some users as the primary determinant of whether their training goals have been achieved.

The ability to record lap times during swimming allows for the intensity of effort to be monitored closely during training and to assess progression. Statistically significant differences in lap time measurements were found for all of the four swimming strokes for both the Finis and the Garmin monitors, with the devices overestimating the time to complete laps (Table 2). Ultimately, statistically significant differences in lap times may not be very relevant to a recreational swimmer, who may be satisfied with a close approximation. A two second error over a typical lap time of 25 seconds would represent an error of 8%. Both activity monitors were found to perform within these limits for frontcrawl swimming. However, this was not found to be the case for the other three strokes. The average error in frontcrawl lap time was 0.58s and 1.09s for the Finis and Garmin monitors, respectively, over an average lap time of 21.24s (i.e. 7.7% and 8.4% error). The maximum lap time error was found for the butterfly stroke (20.6%). Additionally a large range of errors was found for all strokes.

By examining laps at the start, middle and end of intervals, it was found that statistically significant errors, found for the Finis and Garmin monitors could be attributed to the an overestimation in the time taken to complete the first and last laps in a given interval, whilst the middle laps were found to accurately reflect the actual lap time (Fig 2). This finding is consistent with previous research [10].

There are several factors which may help to explain the errors found in the lap times, which averaged over three seconds in some cases (Table 2). A strong push-off and finish are required to detect these events in order to maximise the accelerometer amplitude at impact [27]. Movement that occurs prior to wall push off may have caused the sensor to begin recording a new lap before it had actually begun. For example, a swimmer may position themselves underwater with their feet against the wall before initiating hip and knee extension, resulting in an overestimated lap time. A similar scenario may also occur during rest intervals. Another legitimate concern is that these issues and resultant errors would be further exacerbated when the activity monitors are used by recreational swimmers. Finis’ documentation recommends that the swimmer should remain static during rest intervals and that rest should be at least three to five seconds in duration to avoid the algorithm from registering a turn [28]. This raises an issue of practicality if the swimmer drinks from a bottle or adjusts their goggles during this time, for example. The Garmin monitor requires the user to manually pause and restart the timer to record intervals. This may result in an inevitable overestimation of first and last laps. The Finis monitor features automatic interval detection, but this was not found to lead to improved accuracy, but clearly is more convenient for the swimmer.

It should be noted that if the test protocol had included longer intervals then a greater proportion of the laps performed would have been mid swimming laps, which were found to be accurately registered by both activity monitors. This would have reduced the impact of the starting and ending laps on the overall statistical results. For example, in a 100m interval swim, half of the laps performed are mid swim laps. However, in a 400m interval, these laps would comprise 87.5% of the total laps performed. For recreational swimmers who chose to swim in a continuous manner, without taking frequent rest intervals, this would greatly improve the performance of the activity monitors during their swim. Swimming strokes can be identified from an accelerometer output as regularly occurring peaks in the signal signature, with local maxima and minima tracked and counted [10,13]. The activity monitors tested in the present study were found to perform quite similarly for the stroke count measure (Fig 2). Both monitors showed significant differences from the criterion in stroke count on all but two occasions. The Finis was found to be significantly related to the criterion measure during backstroke only, whilst the Garmin monitor was significantly related for frontcrawl only. Outliers increased the spread of stroke count errors considerably for both activity monitors. Additionally, both activity monitors tended towards overestimation of the stroke count in all strokes except butterfly. The maximum reported error was found to be -7 strokes for the Finis and +7 strokes for the Garmin. That said, both monitors reported the stroke count to within one of the actual stroke count on over 62% of instances.

Previous studies have determined stroke count from either the back, wrist or head [9]. The tendency towards over estimation of the stroke count may be explained by an analysis of the action of the arm on which the sensor is placed. It is standard practice to only record full stroke cycles when determining stroke count. In frontcrawl and backstroke swimming, this means that both the left and right arms must complete a stroke for a cycle to be counted. The algorithms used by these devices record the movements of only one arm however, and multiply this by two to arrive at the stroke count [29]. Therefore, the activity monitors may report an incorrect stroke count depending on which arm is used for the first and last strokes of a given lap.

This would not explain the results for the short-axis strokes however. One possibility for the overestimation in breaststroke stroke count is that the arm action during the push-off and glide phase were erroneously counted as stroke cycles. Variations may also be due to action of the arms before and after a turn. It has previously been suggested that the first and final strokes of a given length can be difficult to record and are more prone to error than strokes performed mid-pool [27]. In butterfly, a swimmer will aim to finish the final stroke with their arms at full extension and as close to the wall as possible. This action may interfere with the stroke count algorithm as the signal may be distorted with the accelerations produced by the turning action of the swimmer. Again like other parameters, we would expect that these errors would be greater when the devices are used by recreational swimmers.

Some of the issues with accuracy may arise from the wrist worn position of these devices. Consistent coordination between left and right arms or upper and lower limb actions cannot be guaranteed. Several studies have objectively demonstrated that variations in inter-arm coordination exist in swimming owing to various factors including swimming speed [22,30]; arm dominance [31]; physical disability [32]; energy cost [33]; exercise intensity [34] and skill level [30]. Furthermore, a similar variance exists between the coordination and synchronisation of the arms and legs for all swimming strokes [23,35]. All of these factors have implications for the accuracy of feature detection algorithms when using wrist mounted devices.

In the present study the average speed over a given length of the pool was determined by both the Garmin and Finis monitors by dividing the pool length (25m) by the time taken to complete each lap. Consequently it is unlikely that this parameter would be of interest to a recreational user as the lap time data would provide a sufficient metric. Ultimately, as a consequence of both activity monitors’ inaccuracies in recording lap times; the results for speed are also significantly different (Table 2). This approach has been evaluated previously and found to overestimate speed [27]. An explanation for this lies in the effects of increased speed following the wall-push off when measured over the full pool length. It is more common in coaching practice for swimming speed to be measured over shorter distances to remove the influence of increased speed during wall push-off. This approach has been found to produce measures of average speed within 3.5%– 4.0% of the criterion values using inertial sensor based systems [12,36]. In the present study the mean absolute percentage error was found to be higher than this, ranging from 7.3% to 16.4%. This can be explained by the issues with the method of determining speed and also by the influence of poorly timed starting and ending laps in a given interval. Again like other parameters, we would expect that these errors would be greater when the devices are used by recreational swimmers.

Stroke rate is the number of strokes a swimmer takes per minute. A typical stroke rate during frontcrawl swimming would be between 35–50 strokes per minute. In comparison to the criterion measure, it was found that the Finis *Swimsense* significantly underestimated stroke rates for all four strokes (Table 3). The average differences ranged from -2.5 strokes per minute (breaststroke) to -9.9 strokes per minute (butterfly), resulting in a maximum expected error of 9.0% and 20.8% error, respectively.

Although specific details of the Finis algorithm are unclear, one possibility is that stroke rate is derived from the stroke count measurements, using the time taken to complete all strokes for a given lap. If this is the case, stroke rate can fluctuate during a lap so is highly dependent on when and how it is measured. In the present study, stroke rates were calculated from the video data using the standard method of measuring the time taken to complete three mid-pool stroke cycles [37]. This difference may go some way towards explaining the underestimated stroke rates registered by the Finis *Swimsense*. Secondly, the accuracy of stroke rate determination depends on the accuracy of the stroke count algorithm, which was found to be error prone. Additionally, small discrepancies in stroke count can lead to large changes in derived stroke rate. For example, if a lap of frontcrawl is completed in 21 seconds and the swimmer completed nine strokes in this lap then the stroke rate would be calculated as 25.7 strokes per minute. However, if the stroke count was overestimated by just one stroke to ten, then the stroke rate would be increased to 28.6 strokes per minute.

Garmin’s documentation suggests that stroke rate is a built-in function of the device but the data provided were the average strokes per minute for the entire swimming session [38]. This information may be of benefit if swimming the same stroke throughout the entire session but not if changing strokes frequently and so is of little value in competitive settings.

The Finis activity monitor determines stroke length by dividing the length of the pool by the stroke count completed by the swimmer in one lap. However this method will overestimate the actual stroke length for the swimmer due to the influence of the wall push off and glide and has been recognised as an unsuitable methodology for some time [39,40]. To illustrate, if ten strokes were completed in a given 25m lap, then the stroke length would be calculated as 2.5m using the Finis algorithm. However, it is typical that the swimmer would have pushed off from the wall and glided for several meters before initiating arm movements. As a result those ten strokes would actually be completed over a shorter distance. If, for example, the swimmer glided for five meters then the actual stroke length would be 2.0 meters. This has shown to be the case as the Finis result revealed a statistical difference with actual stroke length.

A more typical method of calculating stroke length (SL) is to using the formula SL = V/(SR/60); thus relating it to the speed (V) and stroke rate (SR) measures [27,41]. However, even had a direct comparison been made to calculate the stroke length from video footage using the Finis method, poor accuracy would still have occurred as the stroke count results for Finis were in themselves significantly different.

## Conclusions

This is the first study to assess the accuracy of two commercially available swimming activity monitors; the Finis *Swimsense* and Garmin *Swim*. Both monitors were found to operate with a relatively similar performance level. However, as previously noted, with recreational swimmers there can be a very wide variation in skill level and fatigue, with consequent high levels of variation in swim performance in this group of swimmers. Conversely, competitive athletes display more consistent patterns of movement [23] and thus the results obtained in this study would represent expected best case findings for these devices and it would be reasonable to expect that there would be a significant deterioration in the activity monitors’ performance when used by recreational swimmers.

Stroke identification and swimming distance were determined with high accuracy. This feedback alone is likely to be suitable for the majority of recreational swimmers seeking health benefits from swimming. For a recreational user, high precision in lap time measurements is not necessary. It is also important to note that issues with lap time measures are specific to laps performed at the beginning and end of a swimming interval and that lap times performed in the middle of an interval (i.e. during a lap that involves two turns) were measured accurately by both devices. Moreover, issues related to the accuracy of the lap time function are skewed due to the short intervals performed in this study. Improved overall accuracy in lap time measurements can be expected for longer distance swimming intervals.

These activity monitors are designed to be used by swimmers who do not have any means of recording this information or for monitoring trends in performance over time. Consequently, whilst this study has revealed statistical issues related to their performance, both devices offer the recreational user a new way of comprehensively monitoring their physical activity whilst swimming. Future research could aim to evaluate the performance of these devices with this specific cohort of swimmers, to assess how increased variability in stroke mechanics would affect the results. Ongoing developments by the manufacturers of both of these monitors are likely to address these issues, in what is a rapidly expanding area of both research and commercial exploration. Rigorous testing is also necessary to ensure that the devices offer a valid and reliable means of monitoring swimming performance. Such improvements would also increase their applicability for competitive swimming environments.
